# Quality of Life and Its Association With Time in Range Among People With Type 2 Diabetes Mellitus Following Different Dietary Interventions: A Crossover Clinical Trial

**DOI:** 10.7759/cureus.57624

**Published:** 2024-04-04

**Authors:** Aditi R Deshmane, Arti Muley

**Affiliations:** 1 Clinical Nutrition, Indian Institute of Food Science and Technology, Aurangabad, IND; 2 Nutrition and Dietetics, Symbiosis International (Deemed University), Pune, IND

**Keywords:** time-in-range, time restricted intermittent fasting, continuous calorie restriction, continuous glucose monitoring metrics, type 2 diabetes mellitus, dietary intervention, quality of life

## Abstract

Background

Quality of Life (QoL) is an essential consideration in healthcare. Numerous studies have examined QoL in India; however, data on QoL following different dietary interventions are lacking. Similarly, the use of technology such as continuous glucose monitoring (CGM) for diabetes care has independently demonstrated improvements in glycemic control; however, its association with QoL remains limited.

Purpose

The purpose was to study the role of different dietary interventions on QoL and its association with Time in Range (TIR), Time Above Range (TAR), and Time Below Range (TBR) among the Type 2 Diabetes Mellitus (T2DM) population.

Methodology

A crossover interventional clinical trial (CTRI/2022/07/044356) was conducted among participants with T2DM of less than 5 years' duration, aged between 25 and 55 years, with an HbA1c level of less than 8%, and who were on Metformin only. Their QoL was assessed after following two diet patterns: the Continuous Calorie Restricted Diet (CCRD) - calorie reduction with small frequent meals, and Time Restricted Intermittent Fasting (TRIF) - calorie reduction with only two meals a day, using the Modified QoL (MDQOL-17) questionnaire. The association between post-dietary interventions QoL and TIR was studied using a 14-day CGM device.

Results

The overall QoL of 51 participants at the end of the dietary interventions was significantly better compared to their QoL before any dietary intervention (85.6±19.0% and 63.1±13.0%, respectively, p = 0.000). Decreased TIR correlated with increased role limitations due to physical functioning (p = 0.002) and decreased energy levels (p = 0.00). As TBR increased, role limitation due to emotional well-being increased, and energy levels decreased significantly (p = 0.01). As TAR increased, energy levels decreased (p = 0.01). A simple linear regression model was statistically significant for role limitations due to physical functioning (p = 0.003) and energy fatigue (p = 0.000), suggesting that higher TIR is associated with higher scores in these domains.

Conclusion

Dietary interventions that improve the TIR and reduce the TAR and TBR can enhance the QoL of individuals with T2DM.

## Introduction

Chronic diseases such as cancer, heart disease, stroke, diabetes, bowel diseases, renal disease, and CNS diseases have been shown to significantly impact an individual's Quality of Life (QoL) [1]. Among all chronic diseases, Type 2 Diabetes Mellitus (T2DM) is a global health concern due to its increasing prevalence [2] and affects health-related QoL the most. Uncontrolled blood glucose levels, the presence of diabetes-related complications, a constant need for self-care, lifestyle adjustments, dietary choices, daily routines, and potential modifications in work routines or living environments profoundly impact QoL in individuals with diabetes. It affects their daily functioning, emotional well-being, and social interactions [3]. Therefore, achieving and maintaining optimal glycemic control, along with weight management and preventing complications, are primary goals; however, the impact of the disease on an individual's QoL is equally significant.

QoL is a multidimensional concept that reflects an individual's perception of their well-being and satisfaction in different areas of life. Assessing QoL involves evaluating different domains, including physical functioning (mobility, daily activities, functional capacity, and pain), emotional state (psychological well-being, encompassing mental health and emotional stability), social interactions (maintaining healthy relationships, having access to supportive social networks), and the ability to pursue meaningful activities (employment and productivity). Therefore, QoL goes beyond the absence of illness or disease and focuses on the individual's ability to enjoy life and experience a sense of fulfillment, an essential healthcare consideration [4].

Different dietary interventions, by their multifaceted impact on immunomodulatory, molecular mechanisms, and other biological impacts, offer valuable insights into the potential application of these dietary interventions as adjunctive therapies in disease management [5]. By adopting appropriate dietary interventions, individuals with T2DM can significantly improve their QoL and reduce the burden of the disease [6]. Various dietary approaches, including the Mediterranean diet and low-carbohydrate diets, have shown promising outcomes in terms of glycemic control, weight management, and psychological well-being [7-9]. Numerous studies have examined QoL in India [10], but no data are available that investigated QoL scores following diet interventions like the Continuous Calorie Restricted Diet (CCRD) or Time Restricted Intermittent Fasting Diet (TRIF).

Continuous glucose monitoring (CGM) derived measures like Time-in-Range (TIR), Time-above-Range (TAR), and Time-below-Range (TBR) are increasingly relevant for research and clinical practice in assessing glycemic control [11]. The subjective relevance of these parameters, as reported by patients, is still limited, as very few studies, especially in diabetes, have studied QoL and its association with TIR. However, no studies have been conducted for T2DM [12,13]. Hence, we wanted to test the efficacy of the two diets and hypothesized that either of the dietary patterns would have a significant impact on the CGM-derived metrics. The primary objective of this study was to assess the pre-post effect of dietary intervention on QoL and CGM, with the secondary objective to observe the difference in QoL and TIR following the two diets, and lastly, to associate QoL with TIR.

## Materials and methods

Study design

A randomized crossover clinical trial was conducted to study the efficacy of two specific dietary interventions, namely, the CCRD and TRIF, on QoL. It was an open-label dietary interventional study with a duration of six months. Based on the aim of our study, a crossover design was selected as it is considered more effective than parallel group trials since the treatment effect is compared within subjects [14]. The study is prospectively registered with the Clinical Trial Registry of India (CTRI), an online public record system for the registration of clinical trials conducted in India, housing approximately 61,750 trials as of January 23, 2024.

Study population

The inclusion criteria were set for individuals of any gender, with T2DM between 25 and 55 years of age, having a small diabetes duration (≤5 years), an HbA1c level of 6.5% to 8%, and not on any medications other than metformin. The reasons for keeping these criteria is that the age and duration of disease increase, there are chances of other complications that might need more rigid or complex intervention. Similarly, an HbA1c level of more than 8% would necessitate glucose-lowering drugs, which increases the risk of hypoglycemia. The same rationale applies to medications other than metformin.

The exclusion criteria include individuals with T1DM; individuals with T2DM for more than five years; individuals treated with anti-diabetic medicine other than metformin; pregnant or lactating women with diabetes; and people with any major complications, such as diabetic kidney disease (chronic or acute), pancreatitis, liver failure, and a history of unstable angina or myocardial infarction.

Sample size

Based on the results of a pilot study (details provided in prior presentations) with an SD between the two interventions and TIR as the primary outcome, the sample size was calculated. To achieve a 95% power of the study with a Type 1 error rate of 0.05, the sample size was calculated to be 47. Considering a dropout rate of 20%, 56 patients were enrolled.

Intervention 

Two dietary interventions used in the clinical trial are the CCRD - reducing the average daily caloric intake of a person by 500 calories, and involving small, frequent meals with three major meals (breakfast, lunch, and dinner) and three small meals (morning, mid-morning, and evening snacks). The dietary composition of the diet was as per the Indian Council of Medical Research (ICMR) recommendations for individual macronutrient composition in the management of T2DM: carbohydrate (CHO) 55-60%, protein 12-15%, and fat 20-30% [15]. On the other hand, TRIF follows the same dietary composition as CCRD but with time and number of meal restrictions. The eating window was 8 to 9 hours with a fixed number of meals (only two meals a day, brunch and dinner) and fasting for the rest of the time, while the timing of the meals varied according to the individual's daily work routine.

Data collection

**Figure 1:**
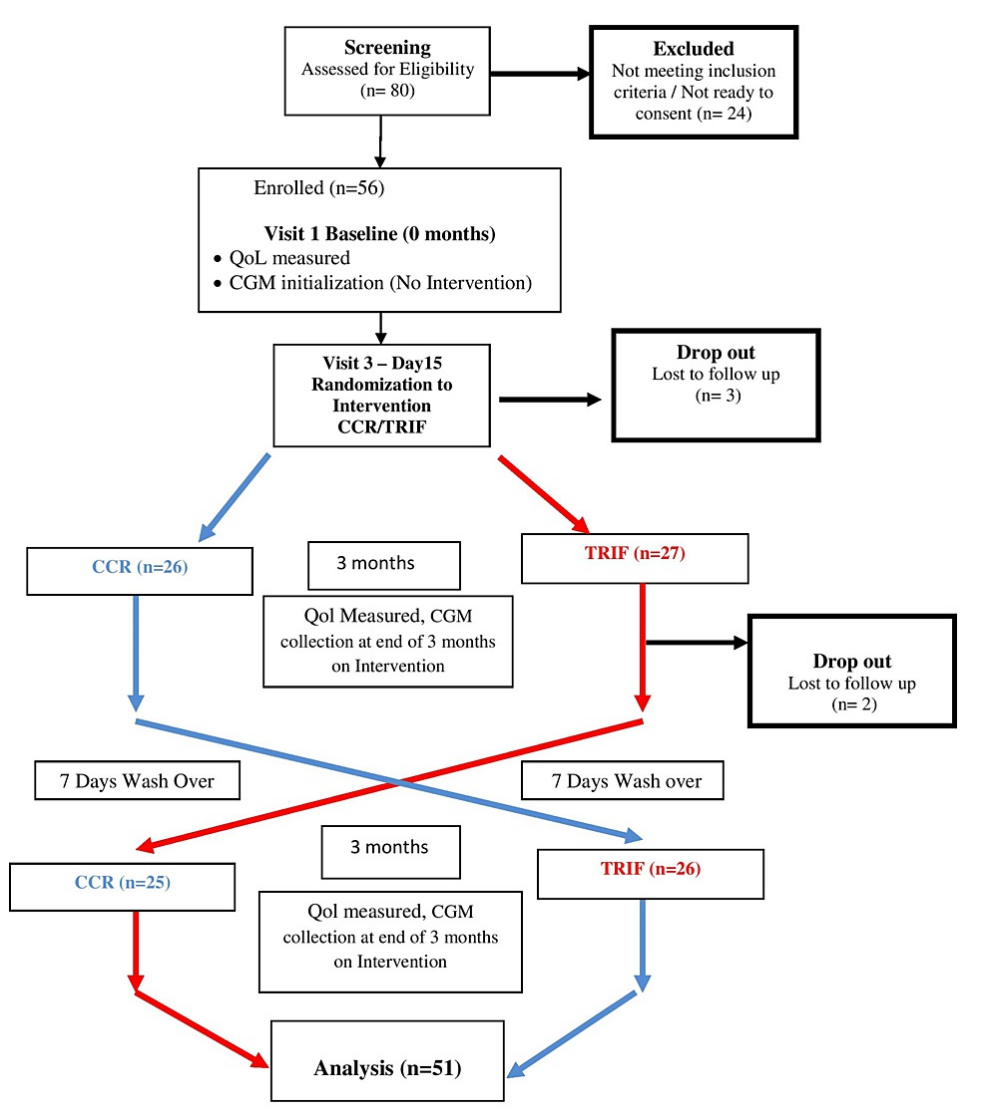
Flowchart of the study design. QoL: Quality of Life; CGM: Continuous Glucose Monitoring device; CCR: Continuous Calorie Restricted Diet; TRIF: Time Restricted Intermittent Fasting.

The two interventions were randomly assigned to the participants using a random block list (block size of 4 and block length of 56). Participants were asked to follow both interventions for three months each, with a washout period of seven days between the two interventions. QoL scores and 14-day CGM data were collected at baseline (before any intervention), at three months (end of the first intervention assigned), and at six months (end of the second intervention assigned). Participants were asked to maintain a food diary, which was used to assess their adherence to the dietary intervention assigned to them. To periodically verify the diet diary, participants were asked to take pictures/photos of their meals and share them with us on random days; these were then compared with the meal recorded in the diary for that day. Additionally, we collected three 24-hour dietary recalls to assess participants' adherence to the interventions. The flowchart of the study design is shown in Figure 1.

Data tools 

QoL data were collected using the Modified Diabetes Quality of Life (MDQOL-17) tool, as this tool has previously been used and validated in the Indian population [16, 17]. This tool consists of domains such as General Health (3 items), Physical Functioning (3 items), Role Limitation due to Physical Health (1 item), Emotional Well-being (3 items), Role Limitation due to Emotional Well-being (2 items), Social Functioning (4 items), and Energy Fatigue (1 item). All 17 items are scored from 0 to 100 and presented as a percentage of the total score. A high score (>70%) is depicted as a good health state, a score between 50 to 70% is considered to represent a moderate health state, and a score of <50% is considered a poor health state. Upon analysis, it was observed that the alpha coefficient for all items of the MDQOL-17 questionnaire was 0.793, suggesting that the items have relatively acceptable internal consistency. Similarly, a FreeStyle Libre Pro CGM by Abbott was used to assess TIR, TAR, TBR. The participants were blinded from the glucose readings of the CGM until the end of the 14-day period.

Study analysis

A total of 51 participants completed both dietary interventions assigned to them, while 5 participants were lost to follow-up. One-way repeated measures ANOVA was used to assess the QoL within the participants following the two dietary interventions as well as CGM-derived metrics. A paired T-test was used to assess the difference in QoL and CGM metrics between CCRD and TRIF. Similarly, correlation and regression analysis were conducted to study the relationship between post-dietary QoL and TIR. Using the procedures recommended by Wellek S and Blettner M [18], carryover and treatment effects were determined at the end of the two intervention trials. All data were analyzed using IBM SPSS version 20. A p-value of <0.05 was considered statistically significant.

## Results

A total of 51 participants completed the trial, of which 33 (64.7%) were male and 18 (35.3%) were female. All participants were categorized as sedentary workers: 14 (27.5%) were housewives, 17 (33.3%) were businessmen, and 20 (39.2%) were professionals or had a job.

At baseline, the QoL scores for all participants were moderate, with a mean score of 63.1 ± 13.0%. About 16 (31.4%) participants had a good QoL with a mean score of 75.7 ± 4.4%, 28 (54.9%) had moderate QoL with a mean score of 61.7 ± 7.9%, and 7 (13.7%) participants had poor QoL with a score of 40.1 ± 5.7%. The overall highest score obtained was 89.18%, while the lowest was 29.24%. In the domain scores, participants scored the lowest in the Energy Fatigue domain with 44.0 ± 19.4% and the highest in the Social Functioning domain with 85.2 ± 20.7%.

A one-way repeated measures ANOVA was performed to compare health-related QoL following dietary intervention among the study population. There was a statistically significant improvement in the overall QoL of participants at the end of both interventions, i.e., at the end of three and six months (85.6 ± 19.0%), compared with the baseline QoL (63.1 ± 13.0%); p < 0.05. A total of 48 (94.1%) participants had a good QoL with a mean score of 82.9 ± 7.0%, while only 3 (5.9%) had moderate QoL with a mean score of 66.4 ± 3.6%. No participant had poor QoL at the end of the study. Like at baseline, participants scored the highest in the Social Functioning domain (94.5 ± 12.8%) and the lowest in the Energy Fatigue domain (68.1 ± 21.6%), post-intervention. There was a statistically significant improvement in all QoL domains. The details of the scores obtained by all participants at baseline and at the end of the dietary intervention study period are shown in Table 1.

**Table 1:** Quality of Life scores obtained by participants at the end of the dietary intervention period. Data presented as mean ± SD, * p-value is significant at <0.05.

Quality of Life Domain	Baseline	End of Intervention 1 (3 Months)	End of Intervention 2 (6 Months)	ANOVA p-value
General Health	53.5 ± 28.1%	69.2 ± 26.3%	79.4 ± 26.6%	0.0001*
Physical Functioning	53.5 ± 35.4%	73.2 ± 26.2%	81.3 ± 24.2%	0.0001*
Role Limitation Due to Physical Functioning	48.0 ± 26.3%	72.5 ± 25.1%	80.3 ± 24.6%	0.0001*
Emotional Wellbeing	63.1 ± 23.7%	85.3 ± 17.7%	90.2 ± 15.4%	0.0012*
Role Limitation Due to Emotional	64.9 ± 27.0%	85.0 ± 18.2%	88.3 ± 16.8%	0.0015*
Social Functioning	85.2 ± 20.7%	92.6 ± 14.5%	94.5 ± 12.8%	0.0001*
Energy fatigue	44.0 ± 19.4%	64.9 ± 23.1%	68.1 ± 21.6%	0.0002*
Total Score	63.1 ± 13.0%	80.1 ± 9.1%	85.6 ± 19.0%	0.0001*

Focusing on improving all domains of QoL implies a shift towards patient-centered care. By understanding an individual's lifestyle, preferences, and goals, healthcare providers can tailor dietary recommendations that are more likely to be sustainable and effective in the long term. Empowering individuals with T2DM through education about the relationship between diet and QoL can be transformative. By understanding how dietary choices impact various aspects of their lives, patients may feel more motivated to adhere to dietary recommendations and take an active role in managing their condition.

**Figure 2:**
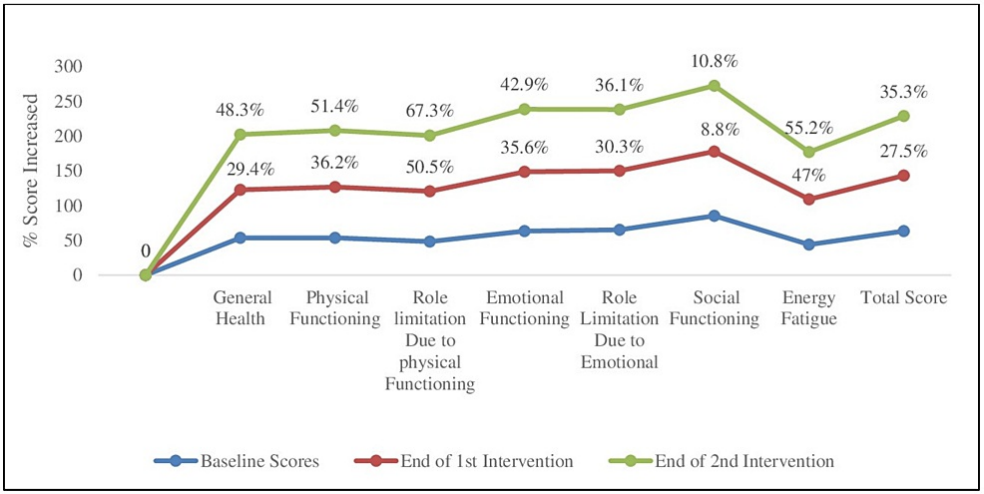
Percent (%) improvement in domain-wise Quality of Life scores compared to baseline.

Looking at the overall change in QoL scores from baseline to the end of each dietary intervention period, there was a total improvement of 35.4% in overall scores at the end of the second intervention compared to baseline. The Role Limitation due to Physical Functioning and Energy Fatigue domains showed the maximum improvement, with 67.3% and 55.2% respectively, compared to the baseline. Improvements in other domains of QoL are shown in Figure 2.

**Figure 3:**
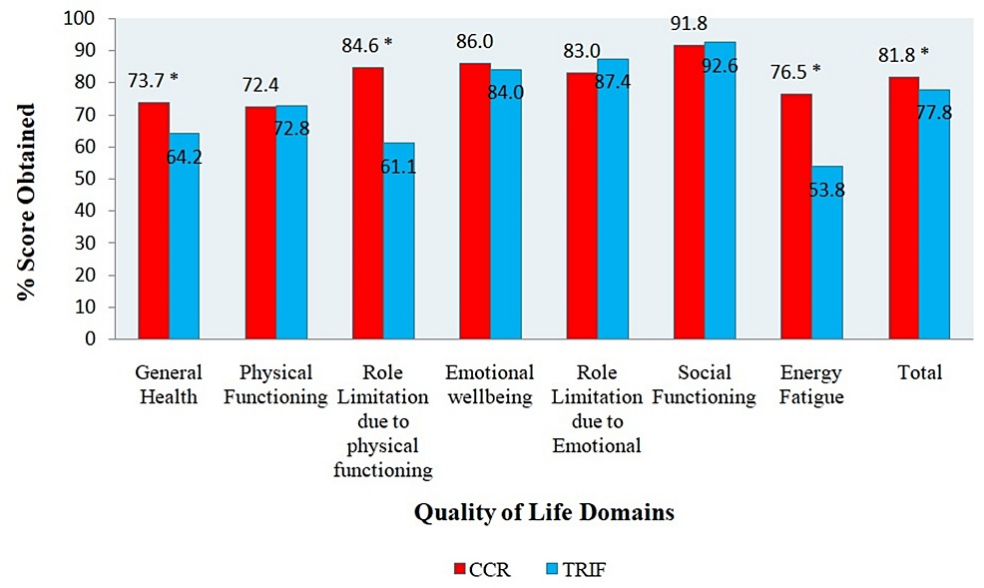
Domain-wise score (%) following two dietary interventions. CCR: Continuous Calorie Restricted Diet; TRIF: Time Restricted Intermittent Fasting. *p-value is significant at <0.05.

A paired sample T-test analysis was performed to examine the presence of any statistical difference in QoL between the CCRD and TRIF within the subjects. The scores obtained by participants are shown in Figure 3. The overall QoL score was statistically better after following CCRD, with a mean score of 84.1 ± 9.7%, compared to QoL scores after following TRIF (mean score 80.1 ± 8.9%, p = 0.04). Similarly, there was a statistically significant difference in domains like General Health, Role Limitation due to Physical Functioning, and Energy Fatigue between the two dietary interventions. Participants following CCRD had better scores in these domains with p < 0.001 than those following TRIF.

A one-way repeated measures ANOVA analysis for the impact of the two dietary interventions on CGM-derived metrics showed that, except for TBR, all other metrics like Average Blood Glucose, Estimated HbA1c, TIR, and TAR were significantly improved at the end of both interventions with a p-value < 0.001 (Table 2). It was observed that Average Blood Sugar levels reduced by 20.2% at the end of the first dietary intervention period and further reduced by 21.4% at the end of the second intervention. Estimated HbA1c levels decreased by approximately 14.6% at the end of both intervention periods. There was an improvement in TIR (by 28% to 30%), and a reduction in TAR (approximately 75%) and TBR (14% to 18%) respectively by the end of the study period.

**Table 2:** CGM-derived metrics at the end of two dietary intervention periods. Data are expressed as mean ± SD; * p-value significant at <0.05. Est A1c: Estimated HbA1c; TAR%: Time Above Range; TIR%: Time in Range; TBR%: Time Below Range.

CGM-derived metrics	Baseline	End of Intervention 1 (3 Months)	End of Intervention 2 (6 Months)	One-way ANOVA p-value
Average Blood Glucose (mg/dl)	151.8 ± 22.6	121.0 ± 19.1	119.2 ± 13.5	0.0001*
Est. A1c (%)	6.79 ± 1.1	5.8 ± 0.6	5.79 ± 0.4	0.0001*
TIR (%)	62.3 ± 5.7	87.0 ± 7.75	86.7 ± 8.34	0.0001*
TAR (%)	31.4 ± 10.3	7.7 ± 7.9	7.8 ± 6.9	0.001*
TBR (%)	6.27 ± 7.0	5.1 ± 5.4	5.4 ± 5.3	0.638

**Figure 4:**
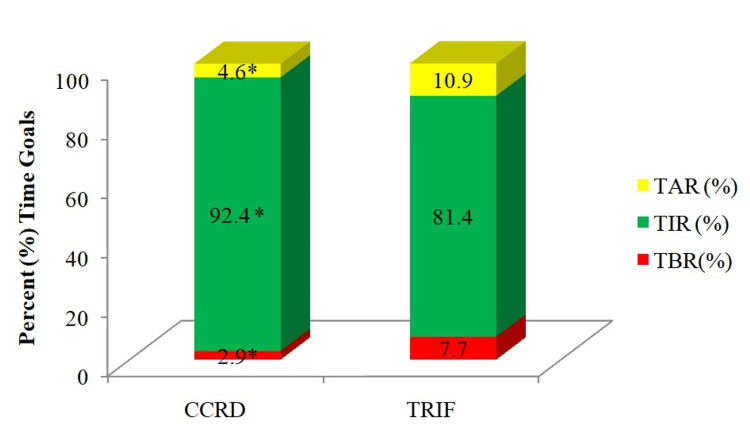
CGM-derived metrics following continuous calorie restricted diet versus time restricted intermittent fasting. *p-value is significant at <0.05. CCRD: Continuous Calorie Restricted Diet; TRIF: Time Restricted Intermittent Fasting; TIR: Time-in-Range; TAR: Time-above-Range; TBR: Time-below-Range.

The difference in CGM-derived metrics following the two diets is shown in Figure 4. Participants had significantly lower TAR and TBR at the end of the CCRD intervention compared to at the end of the TRIF intervention (p < 0.05). On the other hand, TIR was significantly higher at the end of the CCRD intervention compared to that at the end of the TRIF intervention (p < 0.05). This suggests that CCRD has a more favorable effect on CGM-derived metrics compared to TRIF among the participants.

Lastly, we also investigated the association between the post-intervention QoL scores and CGM-derived metrics like TIR, TAR, and TBR. The details are shown in Table 3. Different health-related factors exhibit varying correlations in terms of strength and direction. Compared to TAR, TIR, and TBR appear to have larger and more meaningful correlations with specific factors.

**Table 3:** Association of post-dietary intervention Quality of Life (QoL) domain-wise scores with Time in Range (TIR), Time Above Range (TAR), and Time Below Range (TBR). ** Correlation is significant at <0.01 (2-tailed); * Correlation is significant at <0.05 (2-tailed). QoL: Quality of Life; TIR: Time-in-Range; TAR: Time-above-Range; TBR: Time-below-Range.

QoL Domains	TIR		TAR		TBR	
	Pearson's Correlation	P-value	Pearson's Correlation	P-value	Pearson's Correlation	P-value
General Health	0.204	0.178	-0.275	0.067	0.108	0.479
Physical Functioning	0.123	0.422	-0.206	0.175	0.124	0.419
Role Limitation Due to Physical Functioning	0.456	0.002**	-0.263	0.081	-0.305	0.035*
Emotional Wellbeing	0.086	0.576	0.026	0.864	-0.158	0.299
Role limitation due to Emotional	0.274	0.068	0.025	0.87	-0.332	0.021*
Social Functioning	-0.137	0.37	0.131	0.392	0.005	0.976
Energy Fatigue	0.593	0.001**	-0.349	0.015*	-0.335	0.02*
Total Score	0.229	0.13	-0.22	0.146	-0.006	0.97

Domain-wise analysis shows that as TIR increased, the scores obtained for the Role Limitation due to Physical Health domain also increased significantly (p-value = 0.002), indicating that higher TIR correlated with fewer Role Limitations due to Physical Health. Similarly, a negative correlation was observed between TBR and both Role Limitations due to Physical Health and Role Limitations due to Emotional Well-being. That is, as TBR increased, the scores obtained for both domains decreased. Therefore, we can conclude that the higher the TBR, the more limitations due to physical and emotional health are seen, with p-values of 0.035 and 0.021, respectively. Additionally, it was observed that as TIR increased, there was a significant increase in the scores obtained for the Energy Fatigue Domain (p = 0.00), and as TAR and TBR increased, there was a decrease in scores for this domain (p = 0.01 and p = 0.02, respectively). Therefore, it can be said that as TIR increases, energy levels increase, while they decrease with an increase in TAR and TBR. No significant correlation was observed between the total QoL score and TIR, TAR, or TBR.

To understand the link between QoL and TIR, TAR, and TBR, a simple linear regression was also conducted, with TIR as a predictor and QoL scores for each domain as dependent variables. The overall regression model was statistically significant (F(2, 49) = 7.757, p = 0.003), suggesting that at least one of the predictors (TIR) had a significant effect on the Role Limitation due to Physical Functioning score. The model explained approximately 22.3% of the variability in this domain of QoL, suggesting that higher TIR values are associated with higher scores for Role Limitation due to Physical Functioning. A similar effect was observed in the Energy and Fatigue domain of QoL (F(2, 49) = 13.238, p < 0.001), indicating that TIR has a significant effect on Energy and Fatigue scores. The model explains approximately 34.2% of the variability in Energy Fatigue scores, suggesting that higher TIR values are associated with higher Energy and Fatigue scores (Table 4).

**Table 4:** Simple linear regression to assess the Time in Range as a predictor of Quality of Life. ** Correlation is significant at 0.01 (2-tailed); * Correlation is significant at 0.05 (2-tailed).

Dependent Variables	Predictor Variable – Time in Range (TIR)		
(QoL domain-wise scores)	Adjusted r^2^	Standardized β coefficient	P-value
General Health	0.018	0.258	0.102
Physical Functioning	0.020	0.199	0.203
Role Limitation Due to Physical Functioning	0.223	0.432	0.003**
Emotional Wellbeing	0.001	0.038	0.807
Role limitation due to Emotional	0.099	-0.178	0.234
Social Functioning	-0.031	-0.121	0.449
Energy Fatigue	0.342	0.543	0.0001**
Total Score	0.025	0.272	0.084

## Discussion

We aimed to assess the impact of dietary intervention on QoL as well as to understand the association between QoL and TIR. Upon investigating the QoL at baseline, it was observed that 30.3% of the participants had good QoL, 57.1% moderate QoL, and 12.5% had poor QoL. These study findings differ from those in Kuwait, where 77.0% of the study population had good QoL [19]. In contrast, a study conducted in India reported that most of the participants had poor QoL [20]. Only one study from Saudi Arabia had findings similar to ours [21]. These differences could be attributed to the methodologies adopted, such as the tools used for data collection, the cutoff points to define the scores, and other factors like the socioeconomic background of the participants. The QoL of our participants at baseline was most affected in domains such as Energy Fatigue followed by General Health, while Social Functioning was least affected. When examining domain-wise QoL findings from other studies, it was observed that general health, financial worries, and diet satisfaction were the domains predominantly affecting QoL, while Role Limitation due to Physical Health and Physical Endurance were associated with a better QoL score [22]. Another study revealed that domains like energy mobility, social burden, anxiety, and worry were significant indicators of poor QoL [23]. These differences again may be attributed to the use of different assessment tools. However, it is clear from our study and the reviewed literature that comprehensive care of people living with Diabetes is needed. Thus, not only maintaining good glycemic control but also improving other aspects of life should be emphasized.

At the end of the dietary intervention study period (either CCRD or TRIF), there was a statistical improvement in the QoL of the participants. The overall QoL score of the participants was good. Our findings that dietary intervention improves QoL scores are supported by a similar study [24]. The dietary interventions studied were low-carbohydrate or low-fat diets for diabetes-specific QoL. Another study conducted in New Delhi, India, also investigated the effect of lifestyle interventions on health-related QoL, concluding that making lifestyle modifications would improve QoL among T2DM patients [25]. Similar findings were echoed by another author [26]. Therefore, we can say that people with T2DM should be advised to follow the dietary intervention recommended by a registered dietician for a better and more favorable QoL.

On comparing the QoL between the two dietary interventions, it was observed that 88.7% of the participants following a CCRD and 92.5% of the participants following a TRIF diet reported good QoL. Some studies suggest that CCRDs are beneficial for reducing cardiometabolic risk factors [27] and for the long-term remission of T2DM [28]. Similarly, a study suggests that Intermittent Fasting may play a role in improving glycemic control in the T2DM population [29]. However, we could not find any studies examining the impact of these diets on QoL. Therefore, we can say that our study is unique, and following any of these two dietary interventions will help improve QoL among the T2DM population. CCRD showed a positive impact on general health, better energy levels, and fewer role limitations due to physical health compared to TRIF. A study investigating the benefits, side effects, and knowledge of Intermittent Fasting among the Saudi population noted common side effects like headache, lethargy, mood swings, and dizziness [30]. Therefore, our results support these findings but from the perspective of QoL.

Lastly, we also investigated the correlation between post-dietary intervention QoL and CGM-derived TIR, TAR, and TBR metrics. We found that a decrease in TIR is associated with a decrease in energy levels and an increase in role limitations due to physical health. Meanwhile, an increase in TBR is associated with an increase in limitations due to emotional factors and a decrease in energy levels, and an increase in TAR decreases energy levels. These findings are supported by a study that found a negative correlation between glycemic variability and diabetes-related QoL [12]. Another study, a sub-study of the RESCUE-Trial, showed that TIR independently correlates with more limitations due to physical, emotional, and vitality problems [13]. However, both studies were conducted with the Type-1 Diabetes population, whereas our study focused on individuals with T2DM. Improving CGM metrics can have several direct and indirect impacts on the quality of life for individuals with T2DM. CGM systems offer real-time feedback on glucose levels, which increases awareness, reduces the risk of hyper- and hypoglycemia-related complications, empowers people to make informed decisions, and allows greater flexibility in their daily activities without compromising glycemic control. As a result, they may experience fewer restrictions and a higher QoL.

To our knowledge, this is a unique study as it examines the impact of two dietary interventions on QoL among people with T2DM. Being a crossover study allowed us to reduce biological variability and control many confounding variables such as gender, status of diabetes at the start of the intervention, and duration of the disease. Similarly, there are not many studies conducted on finding an association of QoL with CGM-derived metrics like TIR, TAR, and TBR in this population. This study provides further evidence that these two dietary interventions can be promoted for use in T2DM management. The results can serve as a handy guide for dieticians to counsel people living with T2DM and recommend adaptable dietary advice. The study has a few limitations: firstly, it was a single-center study, though the subjects were from different talukas or districts. Secondly, while obtaining informed consent, participants were assured that their responses would be confidential and that their honest feedback would be very helpful. However, the possibility of a Hawthorne effect cannot be entirely ruled out. Lastly, as the results are for a very specific population, the results cannot be generalized.

## Conclusions

Improving QoL involves addressing the specific needs and challenges that individuals face in their unique circumstances. It surely requires a holistic approach that considers physical, psychological, and social well-being. It is indispensable for healthcare professionals to recognize the multidimensional impact of the disease and prioritize strategies that address these various domains to improve the QoL of individuals with T2DM. By focusing not only on glycemic control but also on improving TIR, healthcare providers can support individuals in living fruitful and satisfied lives despite their diabetes diagnosis. Treatment strategies and interventions should aim not only to manage symptoms but also to enhance quality of life. Minimizing the treatment burden through effective education, personalized care plans, and simplified treatment strategies can significantly increase longevity and add value to the lives of individuals with T2DM.
